# Dietary protein improves flesh quality by enhancing antioxidant ability *via* the NF-E2-related factor 2/Kelch-like ECH-associated protein 1 signaling pathway in softshell turtle (*Pelodiscus sinensis*)

**DOI:** 10.3389/fnut.2022.1030583

**Published:** 2022-11-10

**Authors:** Hongyan Kou, Junru Hu, Xueting Liu, Lijuan Zhao, Kai Zhang, Xunbin Pan, Anli Wang, Yutao Miao, Li Lin

**Affiliations:** ^1^Guangdong Provincial Water Environment and Aquatic Products Security Engineering Technology Research Center, Guangzhou Key Laboratory of Aquatic Animal Diseases and Waterfowl Breeding, Guangdong Provincial Key Laboratory of Waterfowl Healthy Breeding, College of Animal Sciences and Technology, Zhongkai University of Agriculture and Engineering, Guangzhou, China; ^2^Guangdong Key Laboratory of Animal Breeding and Nutrition, Key Laboratory of Animal Nutrition and Feed Science in South China of Ministry of Agriculture and Rural Affairs, Institute of Animal Science, Guangdong Academy of Agricultural Sciences, Guangzhou, China; ^3^Key Laboratory of Ecology and Environment Science in Guangdong Higher Education, Guangdong Provincial Key Laboratory for Healthy and Safe Aquaculture, Guangzhou, China; ^4^Institute of Modern Aquaculture Science and Engineering, South China Normal University, Guangzhou, China

**Keywords:** amino acid profile, antioxidant capacity, flesh quality, Nrf2/keap1 signaling pathway, protein, softshell turtle

## Abstract

An 8-week feeding trial was performed to assess the influence of a gradient of protein levels (14.38–45.23%) on flesh quality, skin color, amino acid profile, collagen, antioxidant capability, and antioxidant-related signaling molecule expression of the softshell turtle (*Pelodiscus sinensis*). Hardness, gumminess, chewiness, and yellowness values in the plastron and carapace, along with collagen, superoxide dismutase, catalase, total antioxidant capacity, and glutathione peroxidase, all improved with elevating dietary protein up to 26.19%, after which they leveled off. Additionally, total amino acids, flavor amino acids, essential amino acids, and non-essential amino acids in the muscle, as well as the expression of copper/zinc superoxide dismutase, glutathione peroxidase, catalase, manganese superoxide dismutase, NF-E2-related factor 2 were all enhanced by increasing the dietary protein level but not changed by higher protein levels. When dietary protein levels were less than 26.19%, the mRNA expression of Kelch-like ECH-associated protein 1, malondialdehyde, and redness values in the carapace and plastron were reduced, as was the lightness values of the carapace, all of which plateaued at higher protein levels. Using catalase activity and malondialdehyde as the indicators and applying a broken-line analysis, the optimal dietary protein level for *P. sinensis* was inferred to be 26.07 and 26.06% protein, respectively. In summary, an optimal protein input improved turtle flesh quality by strengthening antioxidant capacity in muscle tissue and by regulating the expression of antioxidant-related enzymes *via* the Nrf2/keap1 signaling pathway.

## Introduction

Softshell turtles (*Pelodiscus sinensis*) have garnered mounting attention from researchers because of their delicious taste, and high nutritional and medicinal value. Feed comprises ca. 50–70% of the costs in softshell turtle cultivation (1). Protein, the most expensive nutrient in aquatic animals’ diets, has a prominent impact on maintaining and repairing damaged organisms, as well as the induction of various enzymes, hormones, and antibodies needed for many important bodily functions (2). Inadequate dietary protein leads to slowed or stunted growth (3), while extra dietary protein primarily results in an extra energy cost for improved nitrogenous production, which leads to depressed growth. Therefore, it is imperative to know the protein requirement in the feed for *P. sinensis*.

The flesh quality of cultured aquaculture is drawing greater attention as peoples’ living standards improve (4). Diets with unbalanced nutrition would substantially diminish the flesh quality of aquatic animals, entailing a reduction in nutritional values and more variation in texture parameters (5–7). The flesh quality of a product is linked to its degree of consumer acceptance and its market value (7). Texture is a vital attribute for measuring flesh quality that involves assessing hardness, springiness, gumminess, chewiness, cohesiveness, and adhesiveness (8). Flesh quality is often also associated with other biomarkers, i.e., amino acid profile, body color, and collagen. Collagen is the biomarker of quality in the calipash of softshell turtles and is largely contained in various connective tissues to sustain and defend this organism and its tissues (9). Body color is generally recognized as a crucial parameter that consumers rely upon to estimate how edible an aquatic animal is or its ornamental value.

Antioxidant ability is a pivotal function of aquatic animals’ immune defense system (10). Recent research has uncovered relationships between dietary protein and antioxidant enzyme activity and immunity indexes in whiteleg shrimp (*Litopenaeus vannamei*) (11), bighead carp (*Aristichthys nobilis*) (12), Nile tilapia (*Oreochromis niloticus*) (13), and grass carp (*Ctenopharyngodo nidella*) (14–17). The impact of dietary protein content on the flesh quality of grass carp *Ctenopharyngodon idella* (16) and blue shrimp *Litopenaeus stylirostris* is also reported (18). Although Wu et al. (19) showed the impact of protein and lipid inclusions on the growth, gene expression, and muscle quality biomarkers of *P. sinensis*, the protein levels in their study were limited to 40, 45, and 50%. The protein requirement of aquatic species is affected by many factors, including a feed’s ingredients, the age of cultured animals, and the breeding season, among others (1). Differences in dietary feed ingredients may result in different optimal protein levels. Not surprisingly, using the specific growth rate as the response parameter, dietary optimal protein levels for *P. sinensis* can be reduced to 27.11% as reported by our laboratory (20). Further, dietary protein levels were recently shown to increase the flesh quality of grass carp by enhancing its antioxidant capacity (16, 17). However, whether these dietary protein inclusions have a similar effect on *P. sinensis* remains unknown. Hence, this research was initiated to investigate the impact of various supplemented levels of dietary protein on the flesh quality, skin color, amino acid profile, collagen of the calipash, antioxidant capacity, and mRNA levels of antioxidant-related enzymes regulated by signaling molecules [Kelch-like ECH-associated protein 1 (Keap1); NF-E2-related factor 2 (Nrf2)] in the muscle of softshell turtles.

## Materials and methods

### Animals and experimental diets

Softshell turtles bought from a commercial farm in Guangzhou (China) were allowed to acclimate for 3 weeks. Ingredients were ground into powder, weighed, and mixed fully. Experimental diets were designed according to a previous study (1). Fish meal, wheat meal, and soybean meal were served as the protein sources, with corn oil as the lipid source. The wheat meal was used to adjust the protein levels accordingly. The diets consisted of 14.38, 20.41, 26.19, 32.23, 37.63, and 45.23% (control group) protein, respectively. The feeds were frozen at –20°C. Water was added to the feeds in a 1:1.2 (v/w) ratio to render them dough-like before feeding to the turtles.

### Feeding trial

The turtles were deprived of food for 1 day and weighed before starting the feeding trial. The initial weight was 4.02 ± 0.06 g. The turtles were randomly divided into 18 plastic tanks (30 cm × 18 cm × 22 cm), each holding 15 turtles and including 20 L of water. Each group had three replicate tanks. The turtles were fed twice (9:00 and 17:00) daily. The water temperature was 30 ± 2°C, at pH = 8.1 during the feeding period.

### Analysis and measurements

#### Sample collection

After finishing the feeding trial, the turtles were deprived of food for 1 day and weighed. Muscle from five turtles from each tank was collected and frozen at –80°C for later measurement.

#### Proximate composition analysis of muscle of softshell turtles

Proximate compositions of muscle in the sampled turtles were determined following our previously described methodology (1).

#### Muscle amino acid profile of softshell turtles

Muscle tissue samples of *P. sinensis* were hydrolyzed in a standardized way. The amino acids in muscle were identified and quantified by an Amino Acid Automatic Analyzer (L-8900, Hitachi Ltd., Japan).

#### Measurement of calipash collagen in softshell turtles

The collagen was removed from the calipash of turtles, by following a previous methodology (21). The content of hydroxyproline (Hyp) was estimated by the colorimetric method described in Messia et al. (22). The content of collagen was measured by multiplying the content of 4-hydroxyproline content (g/100 g sample) (23), whereby a ratio of nitrogen to protein of 6.25 corresponds to collagen connective tissue having12.5% 4-hydroxyproline.

#### Muscle and calipash flesh quality parameters

Muscles from limbs were sampled from three turtles per tank. The texture parameters of the turtle flesh, namely its adhesiveness, hardness, cohesiveness, chewiness, gumminess, and springiness, were quantified by an instrumental method-texture profile analysis (TPA) (TMS-PRO, FTC, America). The parameters were determined and assessed by following a previously described methodology (24). The measurement conditions include two consecutive compression cycles at a fixed speed of 30 mm/min, 60% deformation of the original length, and an initial force of 0.1 N.

#### Measurement of skin color

At the end of the experiment, the skin color of four turtles from each treatment was tested separately. Skin color was determined in the afternoon by a portable colorimeter (GEB-104 Pantone Color-Cue). This measurement was performed according to the methodology of Wang et al. (25).

#### Determination of antioxidant capacity

Muscle samples were homogenized on ice with ice-cold salt water (0.65%) at 1:5 (w/v) for 1–2 min. The supernatant solution was centrifuged (1,800 × *g*, 4°C) for 15 min and kept at –20°C. The analysis of muscle glutathione peroxidase activity (GPX), the content of malondialdehyde (MDA), total antioxidant capacity (T-AOC), superoxide dismutase activity (SOD), and catalase activity (CAT), were carried out using commercial analytical kits (Jiancheng Institute of Biotechnology, Nanjing, China).

#### Real-time PCR analysis

The muscle tissue of three turtles collected from each tank was pooled and the extraction of their total RNA was performed using an RNAiso Plus Kit (Takara, Dalian, China) by following the manufacturer’s recommendations. The levels of mRNA expression of *CuZnSOD*, *CAT*, *MnSOD*, *GPX1*, *GPX2*, *GPX3*, *GPX4*, *Nrf2*, and *Keap 1* genes were determined. This analysis using a real-time PCR test used the same primers as in our previous study (10).

### Statistical methods

All data were processed and analyzed in SPSS 19.0 for Windows. Differences among the means of treatments were processed by Tukey’s HSD range test at *P* < 0.05. To assess the relationships between CAT, MDA, and dietary protein content, a broken-line model analysis was used.

## Results

### Muscle proximate composition analysis of softshell turtles

The estimation of protein inclusions in the diets on the proximate composition of turtle muscle is presented in Table 1. Increasing the dietary protein concentration from 14.38 to 26.19% augmented the crude protein content of muscle in softshell turtles, but no significant change ensued for protein levels higher than 26.19%. The amount of moisture, ash, and crude lipid in the muscle of the turtles was not impacted by the dietary protein inclusions.

**TABLE 1:** Impact of a gradient of dietary protein levels on the muscle composition of softshell turtles, *Pelodiscus sinensis* (means ± SD, *n* = 15).

Parameter	Gradient of protein levels (%)					
	14.38	20.41	26.19	32.23	37.63	45.23 (control group)
Moisture (%)	79.13 ± 2.25	77.85 ± 2.33	77.43 ± 1.78	77.25 ± 1.52	77.63 ± 1.64	77.86 ± 1.47
Crude protein (%)	14.25 ± 0.09^a^	15.39 ± 0.11^b^	16.13 ± 0.12^c^	16.18 ± 0.13^c^	16.17 ± 0.11^c^	16.14 ± 0.14^c^
Crude lipid (%)	0.66 ± 0.05	0.63 ± 0.06	0.59 ± 0.05	0.61 ± 0.05	0.57 ± 0.05	0.55 ± 0.06
Ash (%)	0.46 ± 0.05	0.47 ± 0.06	0.51 ± 0.07	0.49 ± 0.08	0.52 ± 0.07	0.48 ± 0.06

Values with different superscript letters within a row are significantly different (*P* < 0.05).

### Muscle amino acid profile of softshell turtles

The impact of dietary protein content on the muscle amino acid profile of softshell turtles is shown in Table 2. Increasing the dietary protein to 26.19% evidently led to an increase in essential amino acids (EAAs) in the muscle. But no further change was detected beyond this level. A similar pattern was found for total amino acids (TAA), non-essential amino acids (NEAAs), and flavor amino acids (FAA) of softshell turtles. Dietary protein concentrations negligibly impacted the ratio of EAA/NEAA or EAA/TAA (*P* > 0.05).

**TABLE 2:** Impact of a gradient of dietary protein levels on the muscle amino acid profile of *P. sinensis*, a softshell turtle (means ± SD, *n* = 15).

Amino acids (%)	Gradient of protein levels (%)					
	14.38	20.41	26.19	32.23	37.63	45.23 (control group)
EAA	28.14 ± 0.09^a^	28.95 ± 0.10^b^	29.89 ± 0.11^c^	30.09 ± 0.12^c^	30.11 ± 0.13^c^	30.08 ± 0.12^c^
FAA	28.07 ± 0.07^a^	28.59 ± 0.08^b^	29.48 ± 0.10^c^	29.51 ± 0.11^c^	29.53 ± 0.12 ^c^	29.53 ± 0.11 ^c^
TAA	62.61 ± 0.18^c^	64.33 ± 0.19^b^	66.59 ± 0.19^a^	66.83 ± 0.20^a^	66.86 ± 0.21^a^	66.87 ± 0.22^a^
NEAA	34.51 ± 0.10^a^	35.38 ± 0.12^b^	36.72 ± 0.16^c^	36.74 ± 0.17^c^	36.75 ± 0.15^c^	36.77 ± 0.18^c^
EAA/NEAA	0.82	0.82	0.81	0.82	0.82	0.82
EAA/TAA	0.45	0.45	0.45	0.45	0.45	0.45

Values with different superscript letters within a row are significantly different (*P* < 0.05). EAA, essential amino acids; FAA, flavor amino acids; TAA, total amino acids; NEAA, non-essential amino acids.

### Collagen in the calipash of softshell turtles

**FIGURE 1:**
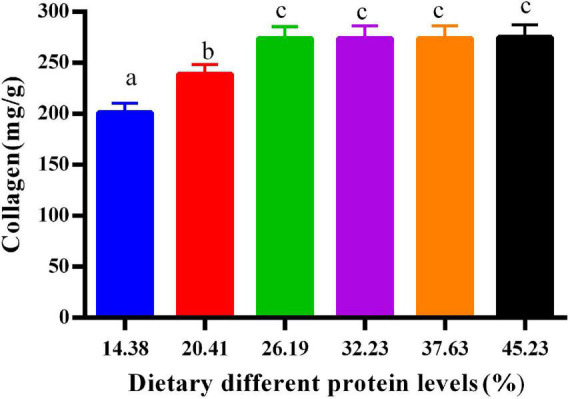
Collagen in the calipash of juvenile softshell turtles fed a gradient of dietary supplementary protein levels for 8 weeks. Bars with different letters indicate significant differences among the groups (*P* < 0.05). The values shown are the mean ± SD for three replicates.

Figure 1 shows the effects of protein on the collagen in the calipash of softshell turtles. The collagen increased sharply with more protein in the diet until the latter reached 26.19%. When dietary protein levels ranged from 26.19 to 45.23%, the collagen in the calipash remained fairly constant.

### Calipash and muscle flesh quality parameters

**FIGURE 2:**
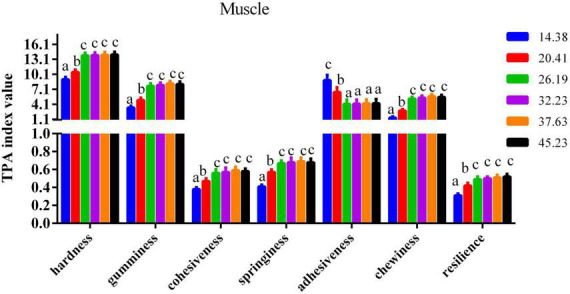
Flesh quality of the muscle from juvenile softshell turtles fed a gradient of dietary supplementary protein levels for 8 weeks. Bars with different letters indicate significant differences among the groups (*P* < 0.05). The values shown are the mean ± SD for three replicates.

**FIGURE 3:**
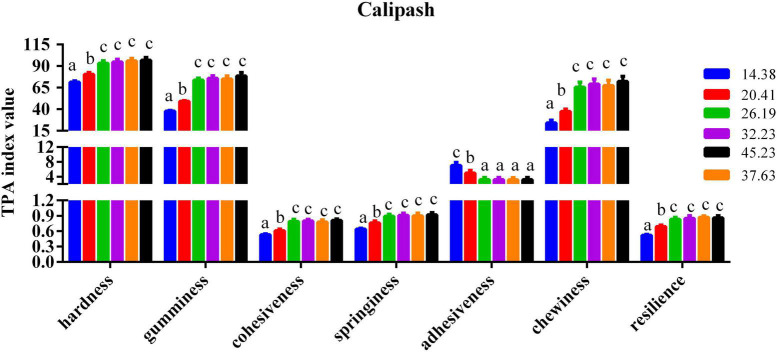
Flesh quality in the calipash of juvenile softshell turtles fed a gradient of dietary supplementary protein levels for 8 weeks. Bars with different letters indicate significant differences among the groups (*P* < 0.05). The values shown are the mean ± SD for three replicates.

The impact of dietary protein inclusions on the various parameters of flesh quality for the muscle and calipash of turtles are shown in Figures 2, 3. The gumminess, chewiness, hardness, cohesiveness, and springiness in the muscle and calipash of softshell turtles were significantly improved when the protein level was raised to 26.19%, after which they were stable. The adhesiveness of both the muscle and calipash of softshell turtles decreased with an increased dietary protein up to 26.19% and was largely unchanged at higher protein contents (*P* > 0.05).

### Skin coloration

**FIGURE 4:**
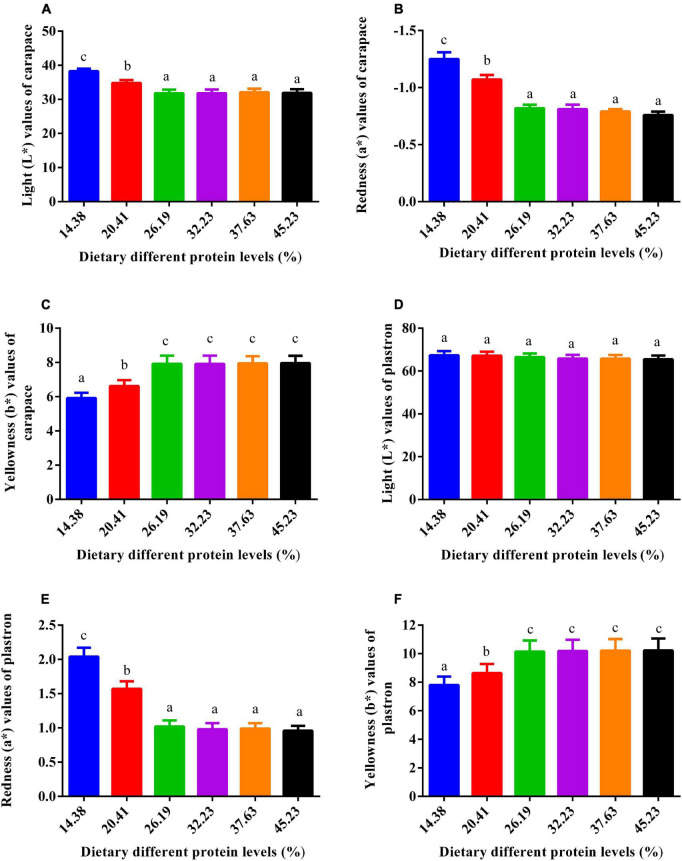
Impact of dietary gradient protein levels on the skin color of juvenile softshell turtles after 8 weeks of feeding. **(A)** Lightness (L*) values in the carapace; **(B)** redness (a*) values in the carapace; **(C)** yellowness (b*) values in the carapace; **(D)** lightness (L*) values in the plastron; **(E)** redness (a*) values in the plastron; **(F)** yellowness (b*) values in the plastron. Bars with different letters indicate significant differences among the groups (*P* < 0.05). The values shown are the mean ± SD for three replicates.

The influence of dietary protein on skin coloration is depicted in Figure 4. Plastron lightness of the six groups presented no pronounced variation (*P* > 0.05). The lightness (L*) and redness (a*) values of the carapace were reduced by higher dietary protein inclusions up to 26.19% after which they leveled off. The trend for yellowness was opposite to that of the redness (a*) and lightness (L*) values. The yellowness values (b*) of the carapace and plastron increased with greater dietary protein level until 26.19%, beyond which they were stable.

### Muscle antioxidant enzyme activity of softshell turtles

**FIGURE 5:**
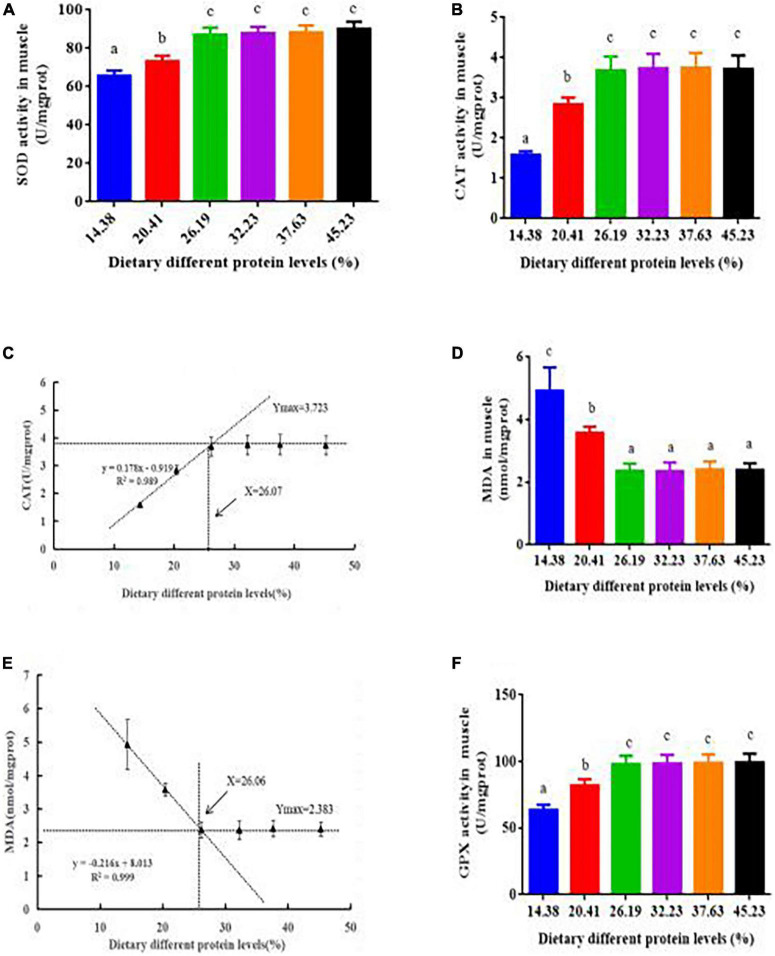
Antioxidant activity in the muscle of juvenile softshell turtles fed a gradient of dietary supplementary protein levels for 8 weeks. **(A)** Superoxide dismutase activity (SOD) and **(B)** catalase activity (CAT). **(C)** Plot presenting the correlation between catalase activity (CAT) and protein levels according to the fitted broken-line model. The breakpoint is 26.07% protein. **(D)** Malondialdehyde (MDA). **(E)** Plot presenting the correlation between malondialdehyde (MDA) and protein levels according to the fitted broken-line model. The breakpoint is 26.06% protein. **(F)** Glutathione peroxidase activity (GPX). Bars with different letters indicate significant differences among the groups (*P* < 0.05). The values shown are the mean ± SD for three replicates.

The activities of SOD, CAT, and GPX rose sharply in response to raising the dietary protein from 14.38 to 26.19% but did not respond further to higher protein concentrations (Figures 5A,B,F). The MDA content declined as the protein level rose from 14.38 to 26.19%, remaining stable when protein levels ranged from 26.19 to 45.23% (Figure 5D). According to the broken-line analysis of CAT and MDA vs. dietary protein levels, their corresponding optimum protein levels were 26.07 and 26.06%, respectively (Figures 5C,E).

### Antioxidant enzyme-related mRNA expression in the muscle of softshell turtles

**FIGURE 6:**
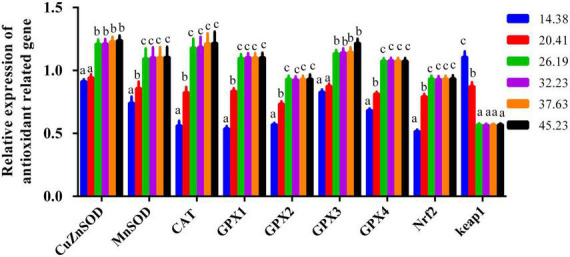
The muscle mRNA expression of antioxidant-related genes of juvenile softshell turtles a gradient of dietary supplementary protein levels for 8 weeks. Bars with different letters indicate significant differences among the groups (*P* < 0.05). The values shown are the mean ± SD for three replicates.

For the genes *CuZnSOD*, *CAT*, *MnSOD, GPX1*, *GPX2*, *GPX3*, *GPX4*, and *Nrf2*, their mRNA expression levels improved with greater dietary protein so long as it was less than 26.19%; adding more protein beyond this level had little apparent effect, as demonstrated by the plateau-like responses (Figure 6). Gene expression of *keap1* presented a trend opposite that of *Nrf2* and was reduced when the protein levels were elevated from 14.38 to 26.19%, staying constant across higher protein levels (Figure 6).

## Discussion

From the results presented, it is evident that dietary protein levels influenced the amino acids, collagen, flesh quality, body color, antioxidant enzymes, and gene expression of antioxidant-related enzymes, Nrf2 and keap1, of the softshell turtle.

Amino acid retention is a key indicator of estimating the amino acid demand of aquatic animals (26). Softshell turtle is popular with consumers due to their high content, and balanced proportion, of amino acids. While EAAs are important amino acids for aquatic animals, FAA is a reliable indicator of tasty food. In our work, increased protein levels augmented the EAA, FAA, and TAA content of softshell turtles. Similar phenomena characterize crabs: when fed a diet with 31.6% protein, they presented lower EAAs and NEAAs than other groups, and the largest EAA and NEAA value was detected in response to 50.2% protein (27). However, dietary protein level did not influence the EAA/NEAA ratio of softshell turtles, whereas crabs supplied with 31.6% protein had a reduced EAA/NEAA ratio relative to other groups (27). This discrepancy may be due to the different aquatic species studied.

In turtles, collagen is a pivotal constituent of hard tissues of the body, such as scales, bones, and the carapace. Therefore, insufficient collagen formation will impair or inhibit the growth of these tissues (28). Collagen is mainly composed of hydroxyl amino acids, which are hydroxylated by lysine and proline (29). From our prior study’s results, both lysine and proline were promoted by more dietary protein to a certain point (1). This may explain why the collagen concentrations were likewise improved with increased protein content up to a certain range. Determining the relative proportion of connective tissue/collagen present is useful for assessing the quality of protein in meat products (30). Our results suggest that a low collagen content ensues when turtles are fed a diet with low protein levels. That is, optimal protein in the diet not only provides balanced nutrition for softshell turtles but also enhances the quality and flavor of their meat.

The texture of the flesh is determined by key freshness quality characteristics including hardness, springiness, chewiness, adhesiveness, cohesiveness, resilience, and the internal cross-linking in the muscle and connective tissue (8). It is generally accepted that hardness is an indispensable texture parameter that indicates the internal cohesion of the flesh. Flesh with low values of hardness generally has loose muscles and is less accepted by meat consumers (31), in part because soft flesh reduces recognition and is associated with low quality. For consumers, firmness of flesh is generally desired (32). According to our results, the gumminess, hardness, and chewiness of the muscle and calipash of softshell turtles increased due to higher protein levels in their feed. High collagen content leads to flesh firmness of Atlantic salmon (*Salmo salar*) (33). The improvement conferred by dietary protein to the hardness of aquatic animals’ flesh may arise from the influence of a suitable dietary protein concentration upon collagen synthesis. Our findings are consistent with a previous report, in which an optimal dietary protein inclusion progressively improved the muscle concentration of hydroxyproline in grass carp (16). This increase may be due to more collagen forming that resulted from the improved protein inclusion in the feed for softshell turtles. With more dietary protein ingested, the hardness of their muscle showed an upward trend, indicating that a higher protein level may improve the intermuscular binding force to render the muscle firmer. This result is consistent with the other findings for *P. sinensis* (19) as well as for *Leiocassis longirostris* Gunther (34). Another reason for this increase may be due to the greater protein content, which can foster tighter intermuscular connections. The correlation analysis of our findings presented a positive relationship between crude protein content in the muscle and *P. sinensis* muscle hardness (*r* = +0.934, *P* < 0.01), chewiness (*r* = +0.951, *P* < 0.01), gumminess (*r* = +0.932, *P* < 0.01), springiness (*r* = +0.955, *P* < 0.01), and resilience (*r* = +0.919, *P* < 0.01), indicating that optimal dietary protein content partly improved the flesh quality *via* elevating crude protein to improve flesh hardness, chewiness, gumminess, cohesiveness, and resilience in turtles. A negative correlation between crude protein and the muscle adhesiveness of *P. sinensis* (*r* = −0.933, *P* < 0.01) was found. Previous studies found that flesh quality can be improved by increasing the muscle’s anti-oxidant ability, which could prevent extra oxidative stress from occurring in the muscle of grass carp (15, 35). This also supports our findings that softshell turtles fed a diet richer in protein had stronger antioxidant capacity.

Body color is not only a pivotal trait for the classification of aquatic species but also a parameter for evaluating their health condition. Softshell turtles have a carapace and plastron, both of which are pliable and soft-shelled. The epidermis of the plastron and carapace is covered with a thick stratum corneum. Similar to other aquatic animals, the pigment cells in softshell turtles are generally distributed under the basal epidermis or in the superficial dermis (36). Although yellowness is one of the unique coloration traits of softshell turtles, the carapace and plastron show distinct coloration patterns: the color of the carapace is yellowish-green, while the plastron is yellowish (25). Softshell turtles with a yellow-like color pattern are recognized as healthier and safer meat products, of higher quality, and even ascribed higher commercial market value (25). Increased protein levels can improve the yellowness and lessen the lightness of the turtles. The mechanism of how the protein levels affect skin color is unclear and should be studied.

Previous papers have shown that antioxidant enzyme levels can serve as indicators of stress and immunological reactions in animals to estimate their general health (25). T-AOC comprehensively reflects the combined antioxidant effects of the enzymatic antioxidant and non-enzymatic systems. It lets one gauge the antioxidant ability of an aquatic organism, while the proportion of MDA can be used to infer the damage that an aquatic organism has incurred as a result of free radical oxidation (37). CAT, SOD, and GPX are pivotal antioxidant enzymes that shape antioxidant resistance to free radical attacks on body cells (38). A decreased flesh quality resulting from nutrition deficiency may be due to oxidative stress in the muscle of aquatic species. Research has indicated that the protein content, muscle hydroxyproline concentration, and protein (amino acid) metabolism may be reduced by protein oxidation (10). These phenomena are in line with findings for other aquatic animals. Tryptophan deficiency can cause muscle oxidative damage, resulting in a lower meat quality of grass carp (*Ctenopharyngodon idella*) (7). In our work, suitable protein levels decreased the oxidative stress and increased the muscle antioxidant capacity, in this way improving the flesh quality of softshell turtles.

Literature showed that the flesh quality of grass carp may be enhanced by elevating the antioxidant ability to prevent excessive muscle oxidative injury (35). Similarly, the correlation analysis of our results demonstrated that the *P. sinensis* muscle hardness was positively associated with SOD (*r* = +0.920, *P* < 0.01), CAT (*r* = +0.948, *P* < 0.01) and GPX(*r* = +0.924, *P* < 0.01); muscle gumminess was positive-associated with SOD (*r* = +0.938, *P* < 0.01), CAT (*r* = +0.920, *P* < 0.01) and GPX (*r* = +0.936, *P* < 0.01); chewiness was positively associated with SOD (*r* = +0.910, *P* < 0.01), CAT (*r* = +0.890, *P* < 0.01) and GPX(*r* = +0.915, *P* < 0.01); cohesiveness was positively associated with SOD (*r* = +0.861, *P* < 0.01), CAT (*r* = +0.936, *P* < 0.01) and GPX(*r* = +0.914, *P* < 0.01); adhesiveness was negatively associated with SOD (*r* = −0.899, *P* < 0.01), CAT (*r* = −0.823, *P* < 0.01) and GPX (*r* = −0.834, *P* < 0.01); resilience was positively associated with SOD (*r* = +0.939, *P* < 0.01), CAT (*r* = +0.960, *P* < 0.01) and GPX (*r* = +0.922, *P* < 0.01), demonstrating that appropriate protein content in the feed may partially increase flesh hardness, gumminess, chewiness, cohesiveness and resilience by increasing the activities of CAT, GPX, and SOD in turtles. The correlation analysis of our findings presented a negative correlation between MDA and the *P. sinensis* muscle chewiness (*r* = −0.922, *P* < 0.01), hardness (*r* = −0.932, *P* < 0.01), gumminess (*r* = −0.894, *P* < 0.01), springiness (*r* = −0.947, *P* < 0.01), cohesiveness (*r* = −0.905, *P* < 0.01) and resilience (*r* = −0.904, *P* < 0.01), indicating that optimal dietary protein content partly improved the flesh quality by decreasing MDA to improve flesh gumminess, hardness, chewiness, cohesiveness and resilience in turtles. In a word, optimal dietary protein content enhanced the flesh quality of *P. sinensis* to some extent through elevating antioxidant ability.

The mRNA expression of antioxidant-related enzymes can reflect the *de novo* synthesis of the antioxidant-related enzymes (6, 7). We found that both the mRNA level and activity of antioxidant-related enzymes in turtle muscle were impacted by dietary protein levels. A previous study proved that Nrf2/Keap1 is a pivotal signaling pathway that keeps the equilibrium between antioxidants and peroxide (24). Other reports revealed that greater *Nrf2* expression could lead to higher mRNA levels of this antioxidant enzyme in young grass carp (7, 35). We found similar findings in the present paper. Increasing protein levels enhanced the mRNA expression of *GPx*, *CAT*, *SOD*, and *Nrf2* in the muscle of turtles, and thus bolstered their flesh quality and collagen content. The increased nuclear translocation of Nrf2 and promoted expression of *CAT*, *GPx1*, *GPx2*, *GPx3*, *GPx4, CuZnSOD*, and *MnSOD* genes caused by optimal dietary protein inclusions might be due to down-regulating *Keap1* gene expression in the muscle of softshell turtle. These findings suggest more *Nrf2* mRNA and less *Keap1* mRNA could improve *CAT*, *CuZnSOD*, *MnSOD*, *GPx1*, *GPx2*, *GPx3*, and *GPx4* mRNA levels in softshell turtles.

## Conclusion

Supplementing feed with optimal protein could increase the growth performance and amino acid content of softshell turtles, in addition to the amount of collagen in their calipash and their skin color and flesh quality parameters. Increased protein levels bolstered the activity of antioxidant enzymes, up-regulated the expression of antioxidant-related genes, and promoted the Nrf2/keap1 signaling pathways, thereby enhancing both antioxidant capacity and flesh quality. These findings will play a prominent role in exploring and elucidating the molecular mechanism responsible for the augmented flesh quality of softshell turtles driven by dietary protein. Therefore, extra or deficient protein in the diet may be harmful to *P. sinensis* and their cultivation. A broken-line model fitted to the measurements of CAT activity and MDA suggests the appropriate protein concentration for softshell turtles is 26.07 and 26.06% protein, respectively.

## Data availability statement

The original contributions presented in this study are included in the article/supplementary material, further inquiries can be directed to the corresponding authors.

## Ethics statement

This animal study was reviewed and approved by the Institutional Animal Care and Use Committee of Zhongkai University of Agriculture and Engineering.

## Author contributions

HK and LL: conceptualization. HK, JH, XL, and XP: investigation. HK and XL: visualization. HK: writing the original draft. HK, LZ, KZ, and AW: writing and editing. YM: supervision. All authors have agreed to the final version of this manuscript.
